# *Periodic flashing* coordinated reset stimulation paradigm reduces sensitivity to ON and OFF period durations

**DOI:** 10.1371/journal.pone.0203782

**Published:** 2018-09-07

**Authors:** Danil Tyulmankov, Peter A. Tass, Hemant Bokil

**Affiliations:** 1 Boston Scientific Neuromodulation, Valencia, California, United States of America; 2 Department of Neurosurgery, Stanford University School of Medicine, Stanford, California, United States of America; Georgia Institute of Technology, UNITED STATES

## Abstract

Pathological synchronization in the basal ganglia network has been considered an important component of Parkinson’s disease pathophysiology. An established treatment for some patients with Parkinson’s disease is deep brain stimulation, in which a tonic high-frequency pulse train is delivered to target regions of the brain. In recent years, a novel neuromodulation paradigm called coordinated reset stimulation has been proposed, which aims to reverse the pathological synchrony by sequentially delivering short high-frequency bursts to distinct sub-regions of the pathologically synchronized network, with an average intra-burst interval for each sub-region corresponding to period of the pathological oscillation. It has further been proposed that the resultant desynchronization can be enhanced when stimulation is interrupted periodically, and that it is particularly beneficial to precisely tune the stimulation ON and OFF time-windows to the underlying pathological frequency. Pre-clinical and clinical studies of coordinated reset stimulation have relied on these proposals for their stimulation protocols. In this study, we present a modified ON-OFF coordinated reset stimulation paradigm called *periodic flashing* and study its behavior through computational modeling using the Kuramoto coupled phase oscillator model. We demonstrate that in contrast to conventional coordinated reset stimulation, the periodic flashing variation does not exhibit a need for precise turning of the ON-OFF periods to the pathological frequency, and demonstrates desynchronization for a wide range of ON and OFF periods. We provide a mechanistic explanation for the previously observed sensitivities and demonstrate that they are an artifact of the specific ON-OFF cycling paradigm used. As a practical consequence, the periodic flashing paradigm simplifies the tuning of optimal stimulation parameters by decreasing the dimension of the search space. It also suggests new, more flexible ways of delivering coordinated reset stimulation.

## Introduction

Parkinson’s disease (PD) is a progressive neurodegenerative disease, the prevalence of which is estimated at 0.3% of the overall population in industrialized countries and advances to 1% by the age of 60 and 4% in the highest age groups [1]. The hallmark signs of PD include movement disorders such as bradykinesia, resting tremors and muscle rigidity [2]. The motor symptoms of PD are associated with a dopamine deficiency resulting from the degradation of dopaminergic neurons in the substantia nigra pars compacta. At present there is no cure for PD; treatment is focused on medical management of motor symptoms. Medical therapy has been primarily focused on restoring dopamine levels through the administration of levodopa, dopamine agonists, or monoamine oxidase B inhibitors [3,4]. Current standards for subject care recommend levodopa as first line therapy for the symptomatic control during the early, uncomplicated stages of PD [3,4]. Unfortunately, chronic treatment with levodopa frequently leads to significant side effects, especially dyskinesias and motor fluctuations [5,6].

Previously, for subjects who had reduced response to medical therapy, pallidotomy (destruction of the globus pallidus) and thalamotomy (destruction of the thalamus) were the only available surgical treatment options [7]. In the 1990s, high-frequency deep brain stimulation (DBS) was demonstrated to be effective in reducing the motor complications of subjects with PD [6]. Since that time, numerous case studies and trials, as well as three recent, large, multicenter, randomized trials have demonstrated the efficacy of this therapy [8–10]. DBS works by chronically delivering a high frequency (120–180 Hz) pulse train (60–200 μs pulse width) through a surgically implanted electrode, most commonly in the subthalamic nucleus (STN) or the globus pallidus internus (GPi). Although the exact mechanisms of action of DBS are still debated, a likely explanation is due to the inhibition of the neuron cell bodies and excitation of axons, which results in modulation of pathological network activity [11]. Further studies have observed that high frequency stimulation suppresses pathological oscillatory behavior in the beta (13–30 Hz) frequency band of the local field potential [12].

**Fig 1:**
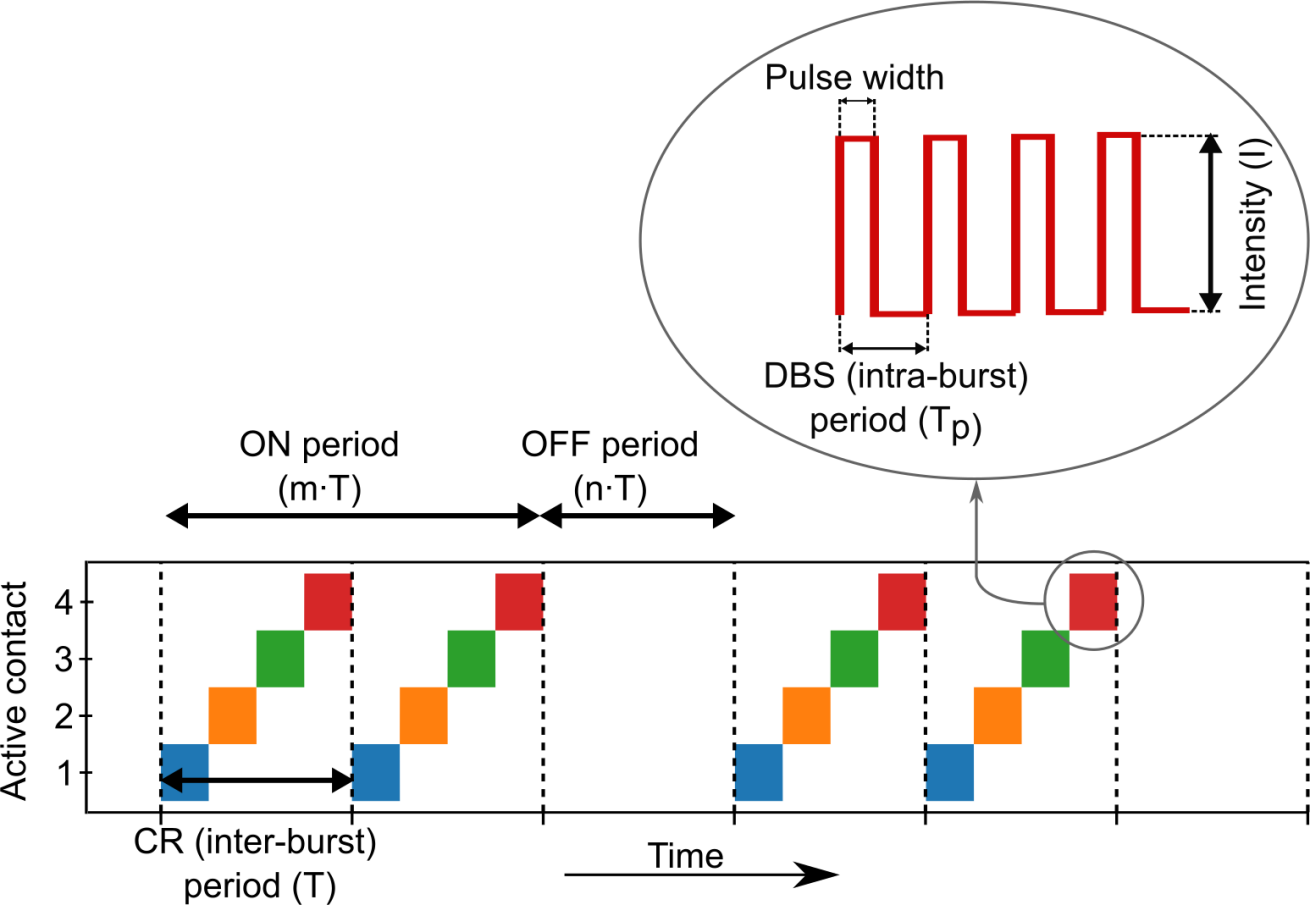
Example coordinated reset stimulation pattern. Bursts of stimulation pulses are delivered sequentially across *N*_*c*_ different stimulation sites (as shown, *N*_*c*_ = 4) distributed along an electrical stimulation lead. Bursts may be delivered in any order within a CR period (e.g. Fig 2). Periods of stimulation (ON) may be interspersed with periods of silence (OFF), with durations defined as integer or non-integer multiples of the CR period (as shown, *m* = 2, *n* = 1). Inset shows an example burst of stimulation, (represented in the main figure as a solid colored block). The number of individual pulses delivered per burst is determined by the intra-burst period and pulse width.

**Fig 2:**
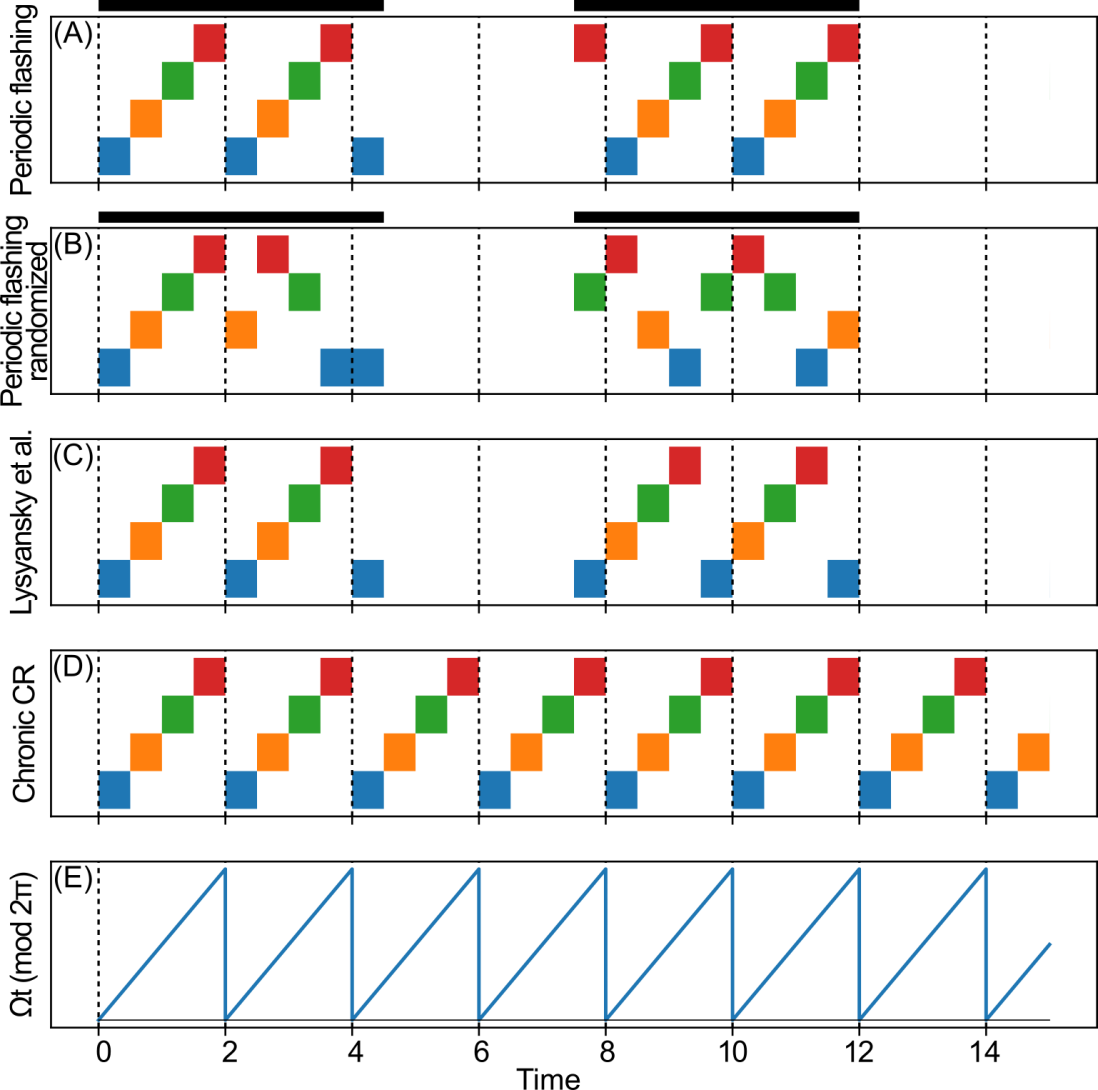
Variations of the coordinated reset stimulation paradigm. (A) Periodic flashing, with fixed sequential contact ordering (*m* = 2.25, *n* = 1.5). Horizontal bars above the plot indicate the ON-OFF “flashing” pattern given by *F(t)*. Note that the activation sites, when active, correspond to the ones that are active during “chronic” CRS in panel D. (B) Periodic flashing, with randomized contact ordering (*m* = 2.25, *n* = 1.5). (C) Stimulation paradigm used by Lysyansky et al. [20] (*m* = 2.25, *n* = 1.5). Note that the stimulation pattern re-starts with stimulation site 1 at every ON cycle. (D) “Chronic” CRS, without ON-OFF cycling. (E) Average phase of the oscillator network (modulo 2π), for reference.

Coordinated reset stimulation (CRS) is a brain stimulation technique predicated on the idea that many neurological disorders, including PD, are caused by pathological synchronization of implicated neural elements. Unlike DBS, which delivers a *tonic* high frequency pulse train, CRS delivers short *bursts* of high frequency stimulation sequentially across more than one electrode of the implanted lead (Fig 1). This pattern of activation, which may include variation in the electrode order (Fig 2), occurs with a frequency that corresponds approximately to the pathological oscillation frequency. Since its proposal in 2003 [13,14], CRS has been refined over the course of many computational modeling studies [15–22], including variations in the electrode order on various time-scales [21], demand-controlled stimulation onset and duration [13–17], and fixed periods with (ON) and without (OFF) stimulation [20].

A major challenge of CRS is finding the optimal parameters for stimulation. Along with the parameters that need to be tuned for traditional DBS–frequency (referred to as *intra*-burst frequency in the context of CRS), intensity, pulse width–it is also necessary to additionally optimize parameters unique to CRS–CR (*inter*-burst) frequency, ON and OFF periods, and sequence of electrode activation (Fig 1). The large number of parameters had made systematic experimental sensitivity analyses difficult. Instead, the preclinical and clinical studies conducted so far have focused on a limited subset of the parameter values, with the intensity, intra-burst frequency and pulse width chosen to be in the standard DBS range, while parameters unique to CRS chosen, in part, based on practical convenience, or on suggestions from computational work [13–22].

First, in epileptic rat hippocampal slice, CRS delivered with 4:2 ON-OFF cycling, at a frequency equal to the mean frequency of the epileptiform discharges, caused long-lasting desynchronization between hippocampal neuronal populations together with a widespread decrease in the amplitude of the epileptiform activity [23]. Next, a study on non-human primates examined the effect of stimulation intensity on the efficacy of CRS. It was observed that CRS delivered for 2 hours/day at the DBS intensity and at one-third of the DBS intensity both provided symptom reduction comparable to that achieved with DBS. In addition, the effect of CRS at one-third of the DBS intensity was long-lasting and persisted for over 1 month after stimulation was turned off. CRS was delivered at a 7 Hz CR frequency with 3:2 ON-OFF cycling [24]. A more recent non-human primate study observed an apparent relationship between the optimal dose and disease severity. A moderate Parkinsonian state required stimulation for 4 hours/day to show symptom reduction comparable to DBS and a mild Parkinsonian state responded more favorably to 2 hours/day than to 4 hours/day. Here again, CRS was delivered at with a 7 Hz CR frequency with 3:2 ON-OFF cycling [25]. Finally, an acute clinical study in 6 patients undergoing DBS treatment for PD demonstrated that CRS delivered for 2 hours, twice a day with 3:2 ON-OFF cycling at the DBS intensity leads to symptom reductions comparable to that seen with traditional DBS [26]. In this study, the intraburst frequency was set to be the peak frequency of the individual patient’s local field potential spectral power.

ON-OFF cycling with periods that are integer multiples of the CRS period has been used in all previous preclinical and clinical studies, raising the question of whether mechanistic reasons exist that make this choice necessary. While using OFF periods to enhance desynchronization was discussed in a number of computational studies [13–17], Lysyansky et al. were the first to examine the effect of varying the ON and OFF periods systematically [20]. In a Kuramoto phase oscillator network model, they demonstrated that the efficacy of CRS is critically dependent on the duration of the ON and OFF periods, a dependence that persists across variations of other model and stimulation parameters. Specifically, the authors demonstrated that by varying the spatial spread and intensity of stimulation, two qualitatively distinct states can be created in the oscillator network. In the “cluster state,” the network is partitioned into subsets of oscillators, each subset having a unique phase offset. In the “desynchronized state,” the oscillator phases become uniformly distributed and achieve uniform desynchronization. Both states exhibit dependence on the ON and OFF periods.

In this work, we introduce a modified paradigm, dubbed *periodic flashing*, in which the timing of the ON-OFF cycles is decoupled from the timing of the stimulation pulse patterns. We study the effect of this paradigm in a phase oscillator network, and demonstrate that this paradigm leads to robust desynchronization for a wide choice of ON and OFF periods. We also show that the dependence seen by Lysyansky et al. is a consequence of the specific CRS paradigm implemented by the authors. In each of the two stimulation regimes discussed above, we establish the mechanism through which the paradigm used by Lysyansky et al. leads to its effects, and detail the reason for failure to achieve desynchronization for certain choices of the ON and OFF period. The periodic flashing paradigm introduced here removes the need to tune two of the CRS parameters–the ON and OFF periods–reducing the search space by two dimensions, potentially simplifying CRS programming and increasing the clinical viability of CRS. It may also open the doors to further enhancement of CRS-like desynchronization approaches.

## Materials and methods

Macroscopically, the collective dynamics of a neuronal network have been previously modeled as an all-to-all coupled network of phase oscillators [13–15,20] as described by Kuramoto [27–29]:

$${\dot{\theta }}_{i}{=\omega }_{i}+\frac{K}{N}\sum_{j=1}^{N}\sin\left(\theta_{j}{-\theta }_{i}\right),\;i=1,2,\ldots ,N$$ #(1)

where *θ*_*i*_ is the phase of the *i*^th^ oscillator, *ω*_*i*_ is the natural frequency of the *i*^th^ oscillator, and *N* is the number of oscillators in the network. The natural frequencies are randomly drawn from a distribution *g(ω)* that is Gaussian with mean *Ω* and standard deviation *σ*_*ω*_. Since all pairs of oscillators are coupled together with the same coupling strength *K*, the spontaneous dynamics of the system (1) are dependent only on the value of *K* and *σ*_*ω*_. If *K* is below the critical coupling strength *K*_*c*_, the oscillators approach an incoherent state where the phases are uniformly distributed on [0, 2π). If *K* > *K*_*c*_, the oscillator phases will spontaneously synchronize. *K*_*c*_ depends only on the distribution of natural frequencies $K_{c}=\frac{2}{\pi g(\Omega )}$ [27–29]. Parkinsonian pathological synchrony is modeled by choosing a value of K above the critical coupling strength (Table 1).

**Table 1:** Definitions and values of model parameters.

Symbol	Definition	Value (if applicable)
*N*	Number of oscillators	200
*K*_*c*_	Critical coupling strength	≈0.0319
*K*	Coupling strength	0.1
*Ω*	Mean of natural frequency distribution of oscillators	π
*σ*_*ω*_	Standard deviation of natural frequency distribution	0.02
*θ*_*i*_*(t)*	Phase of the *i*^th^ oscillator	
*I*	Stimulation intensity	10 or 7
*σ*	Spatial current decay rate	0.4 or 2
*ρ*_*j*_*(t)*	Indicator function if *j*^th^ stimulation site active at time *t*	“Sequential” or “Randomized”
*L*	Length along which oscillators are distributed	10
*x*_*i*_	Location of *i*^th^ oscillator	$\frac{(i-1)L}{N-1}$
*N*_*c*_	Number of stimulation sites	4
*c*_*j*_	Location of *j*^th^ stimulation site	$\frac{(j-\frac{1}{2})L}{N_{c}}$
*D(x*_*i*_, *c*_*j*_*)*	Stimulation impact from contact *c*_*j*_ at location *x*_*i*_	$\frac{1}{1+{\left(\frac{x_{i}-c_{j}}{\sigma }\right)}^{2}}$
*T*	Stimulation cycle length (CR period)	2
*P(t)*	Unit amplitude rectangular wave, period *T*_*p*_, pulse width *T*_*p*_/2	
*T*_*p*_	Inter-pulse interval	1/20
*F(t)*	Indicator function whether stimulation is ON or OFF at time *t*	Periodic with period *(m+n)T*
*m*	Number of CR periods for which stimulation is ON	Varied 1 to 7
*n*	Number of CR periods for which stimulation is OFF	Varied 2 to 7

The degree of synchrony is quantified by computing order parameters of various integer orders. The *m*^th^ order parameter [30,31], where *m* ≥ 1, is defined as

$${Z_{m}=R}_{m}e^{i\psi_{m}}=\frac{1}{N}\sum_{j=1}^{N}e^{im\theta_{j}}$$ #(2)

where 0 ≤ *R*_*m*_ ≤ 1 is the magnitude and *ψ*_*m*_ is the phase. The first order parameter magnitude *R*_*1*_ corresponds to the coherence of the system, and *ψ*_*1*_ measures the average phase. High values of *R*_*1*_ correspond to a strongly synchronized in-phase system with a single phase cluster. Higher order parameters *R*_*m*_, *m* > 1, describe the *m*^th^-order clustering behavior of the system. A high value of *R*_*m*_ combined with low values of *R*_*k*_, 1 ≤ *k* < *m*, corresponds to a state with *m* equally spaced clusters around the unit circle. A fully incoherent state has *R*_*m*_ = 0 for all *m*.

Following Lysyansky et al. [20], we consider the oscillators arranged on a linear segment [0, *L*], located at $x_{i}=\frac{(i-1)L}{N-1},i=1,\ldots ,N$ and *N*_*c*_ stimulation sites located at $c_{j}=\frac{(j-\frac{1}{2})L}{N_{c}},j=1,\ldots ,N_{c}$. To model the CRS paradigm, the stimulation term *S*_*i*_*(t)* is added to the right hand side of (1)

$$S_{i}(t)=I\;\cos\;\theta_{i}P(t)F(t)\sum_{j=1}^{N_{c}}D(x_{i},c_{j})\rho_{j}(t),\;i=1,2,\ldots ,N$$ #(3)

where *I* is the intensity of the stimulation and the cos*θ*_*i*_ term [15] accounts for the phase-dependent effect of electrical stimulation of a single neuron [32,33]. *P(t)* represents a chronic high-frequency stimulation term, here considered as a train of unit amplitude rectangular pulses with inter-pulse interval *T*_*p*_ and pulse width *T*_*p*_/2. The *F(t)* and *ρ*_*j*_*(t)* terms control the ON-OFF and spatiotemporal cycling patterns of the stimulation sites, and *D(x*_*i*_, *c*_*j*_*)* accounts for the spatial profile of the stimulation. We discuss these final three terms in further detail below.

The term *ρ*_*j*_*(t)* is an indicator function that determines whether site *j* is active at time *t*. In its most simple “sequential cycling” variation,

$$\rho_{j}(t)=\left\{ \begin{array}{c} 1,\;(j-1)\frac{T}{N_{c}}\leq t<j\frac{T}{N_{c}}\;(mod\;T)\\ 0,\;otherwise \end{array}\right.$$ #(4)

where each site is activated one at a time, in sequential order, for a duration of $\frac{T}{N_{c}}$, such that each site is activated exactly once during the CR period (Fig 2A). We also consider a “randomized cycling” variation where the order of the activated sites is randomly permuted every CR period (Fig 2B). As done in previous work [13–15,20], the CR period is chosen to be equal to the average natural period of the unstimulated system of oscillators, $T=\frac{2\pi }{\Omega }$.

The term *F(t)* is an indicator function that determines the “flashing” ON-OFF pattern of the entire stimulation; the stimulation is ON for *m* cycles of length *T* and OFF for *n* cycles (Fig 2A and 2B). In contrast to the paradigm used by Lysyansky et al. [20] (Fig 2C), the “flashing” ON-OFF control *F(t)* is independent of the stimulation site cycling *ρ*_*j*_*(t)*. Unlike periodic flashing, the ON-OFF paradigm from Lysyansky et al. enforces the cycling such that site 1 is always the first active site in each ON period.

**Fig 3:**
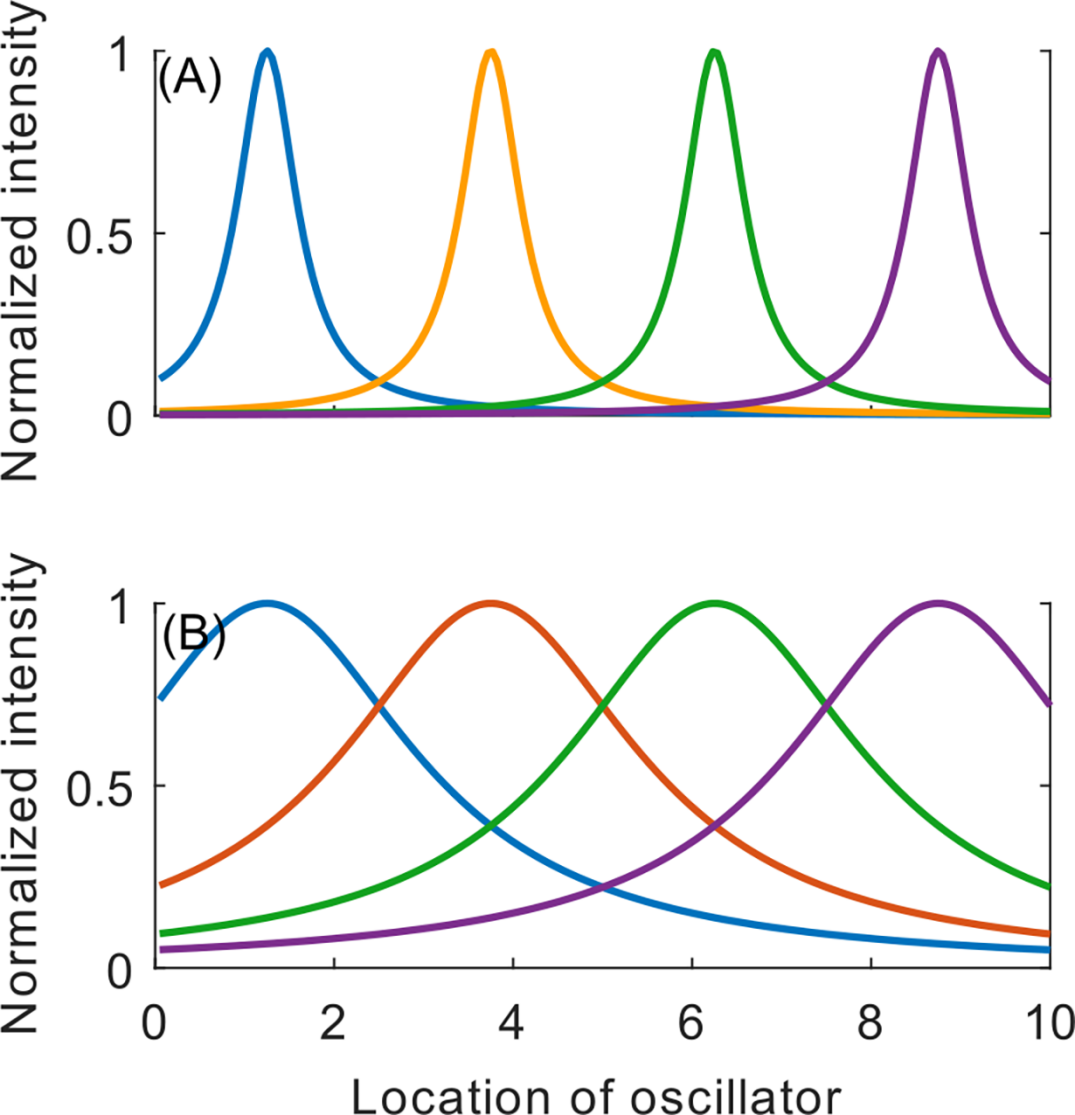
Spatial stimulation decay profile relative to locations of stimulation sites. Normalized intensity profiles as a function of location for (A) σ = 0.4 and (B) σ = 2. Stimulation sites correspond to the peaks of the spatial profiles. Oscillators are evenly distributed along the length of the horizontal axis.

Finally, *D(x*_*i*_, *c*_*j*_*)* is the spatial decay function that describes the impact of stimulation of contact *c*_*j*_ on location *x*_*i*_. As estimated in brain tissue [34] (however, see [35]), the spatial decay is Lorentzian,

$$D(x_{i},c_{j})=\frac{1}{1+{\left(\frac{x_{i}-c_{j}}{\sigma }\right)}^{2}}$$ #(5)

where *σ* defines the spatial decay rate of the stimulation as a function of distance from the contact (Fig 3). Note that although the coupling between the oscillators (1) is independent of their spatial position, the stimulation strength (2,5) depends on oscillator location. The spatial decay function scales the magnitude of the stimulation applied to a particular oscillator as a function of distance from the stimulation site.

See Table 1 for a summary of the definitions and the values used for the parameters in the model.

Finally, to quantify the efficacy of the ON-OFF stimulation, we use the mean maximum of the order parameter [20], defined as

$${\langle r\rangle }_{m}=\frac{1}{N_{F}}\sum_{i=1}^{N_{F}}r_{m}^{\left(i\right)}$$ #(6)

where $r_{m}^{\left(i\right)}$ is the maximum value of *R*_*m*_*(t)* during the *i*^th^ OFF cycle, and *N*_*F*_ is the total number of “flashes,” i.e. ON-OFF cycles. To ensure a steady-state result is reached, we ignore the first 10 flashing periods when calculating *<r>*_*m*_.

We numerically integrate the system described by (1) and (3) using the fourth-order explicit Runge-Kutta method with a step size of 0.001. Although less computationally intensive, we have found that adaptive methods did not produce sufficiently reliable results with the necessary degree of temporal precision.

## Results

**Fig 4:**
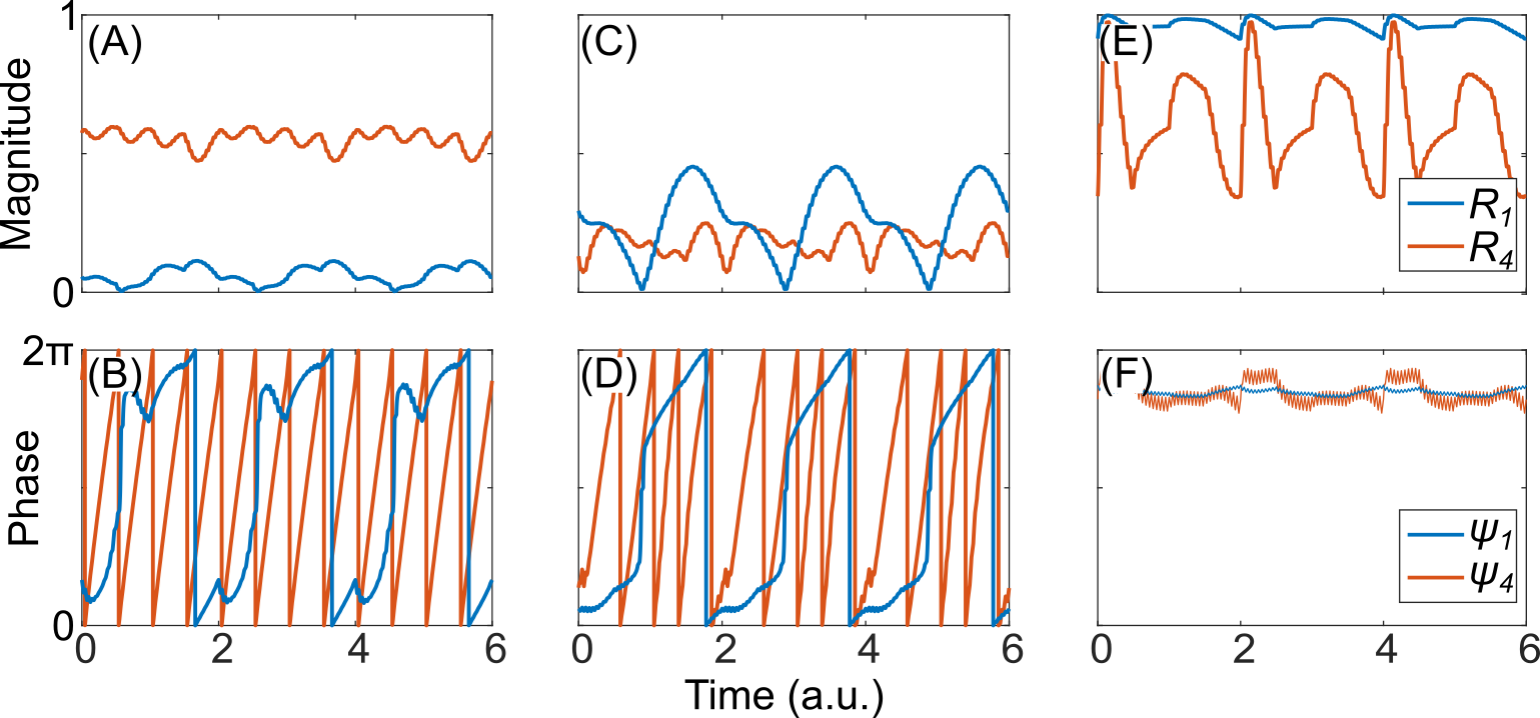
Three parameter regimes achievable by varying stimulation intensity and spatial spread. (A) Magnitude and (B) phase modulo 2π, of the first and fourth order parameter in the “cluster state” regime (*I* = 10, *σ* = 0.4). (C) and (D) in the “desynchronization state” regime (*I* = 7, *σ* = 2). (E) and (F) in the “oscillation death” regime (*I* = 20, *σ* = 4). Note that the phase is approximately constant in the “oscillation death” regime, with a high frequency phase jitter. Only the two parameter regimes shown in subplots A-D are considered in this work.

In this work, we introduce a novel CRS paradigm which we call *periodic flashing*, in which the CR pattern, i.e. the sequence of activation of the stimulation sites, is fixed and operated independently of the ON-OFF “flashing” pattern. We demonstrate that this paradigm is robust to variation of the ON and OFF periods, assuming sufficient stimulation is delivered. Importantly, it is robust for variations of other model and stimulation parameters. Specifically, in the computational work exploring the (*I*,*σ*) parameter space through “chronic” CRS (Fig 2D) Lysyansky et al. [20] noted three distinct states achievable through varying the *I* and *σ* parameters: a “cluster state,” where *R*_*1*_ is low and *R*_*4*_ is high (Fig 4A and 4B), a “desynchronization state” where both order parameters are low (Fig 4C and 4D), and a state of “oscillation death” where both order parameters are high but the phases are fixed and do not change in time (Fig 4E and 4F). Oscillation death is achieved through large values of *I* and *σ*, and is akin to traditional high-frequency DBS which is known to stop oscillation due to high-frequency entrainment [15]. This state is not representative of CRS, and therefore we only focus on two parameter regimes: the “cluster state” regime (*I* = 10, *σ =* 0.4) and “desynchronization state” (*I* = 7, *σ =* 2) regime.

### First-order desynchronization through phase clustering

**Fig 5:**
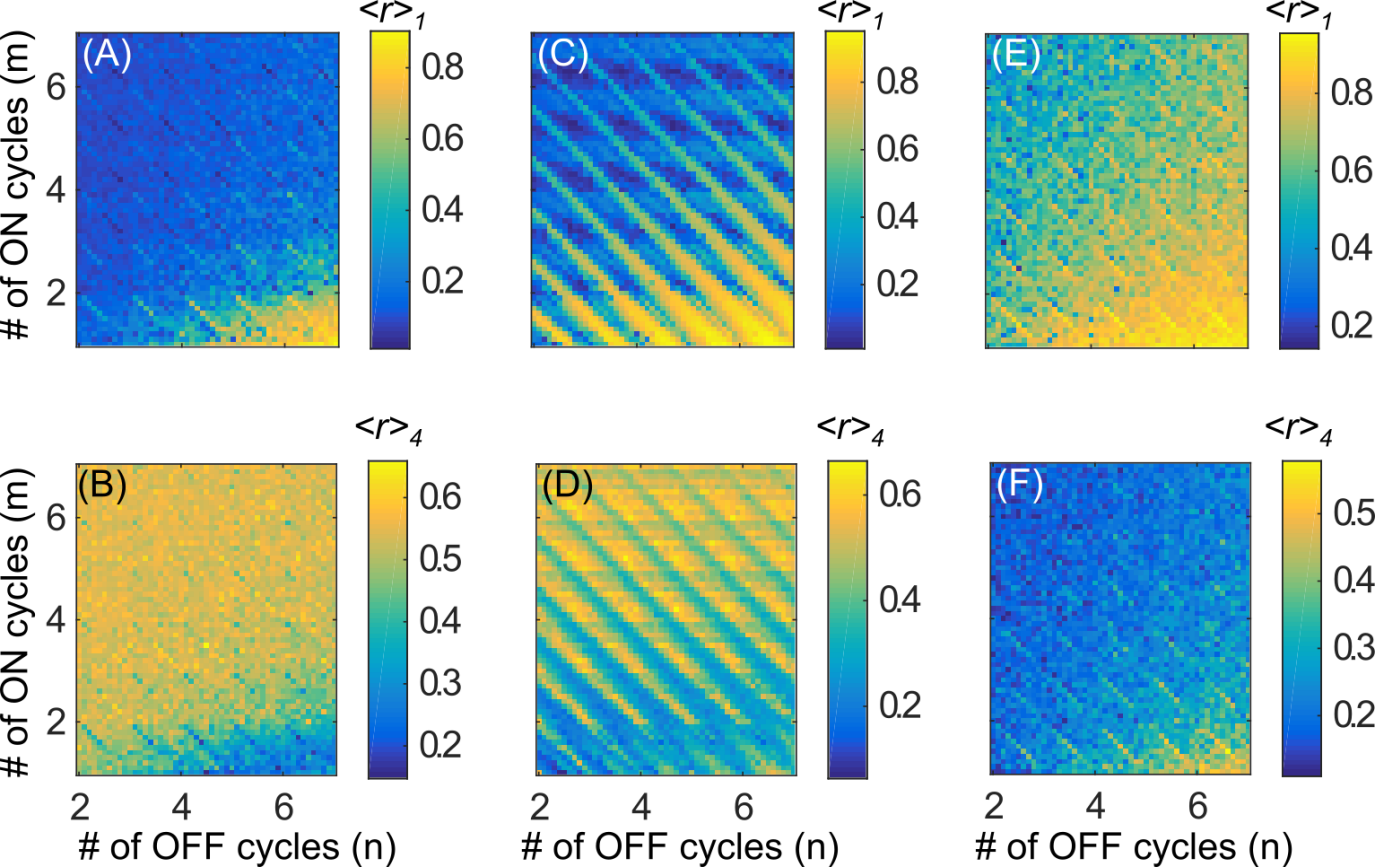
Mean maximum of the order parameter as a function of *m* and *n* for different stimulation paradigms in the cluster state regime (*I* = 10, *σ =* 0.4). (A) *<r>*_*1*_ and (B) *<r>*_*4*_ using periodic flashing, with sequential activation of stimulation sites. (C) *<r>*_*1*_ and (D) *<r>*_*4*_ using the ON-OFF stimulation paradigm from [20]. (E) *<r>*_*1*_ and (F) *<r>*_*4*_ using periodic flashing, with randomized activation of stimulation sites.

We first consider the performance of the periodic flashing CRS paradigm in the cluster state regime. Fig 5A shows the degree of first-order desynchronization achieved by the periodic flashing CRS paradigm as a function of the number of ON (*m*) and OFF (*n*) cycles. It is quantified by the mean maximum of the first order parameter *<r>*_*1*_ (6). Periodic flashing successfully suppresses first-order synchrony in the oscillator network for all values of *m* and *n*, provided that sufficient total stimulation is delivered. In contrast, the CRS paradigm used by Lysyansky et al. [20] only achieves desynchronization for particular choices of *m* and *n* (Fig 5C), specifically where the sum (*m+n*) was an integer, a phenomenon referred to as “anti-resonance”.

To understand why an apparently small difference in stimulation pattern causes qualitative differences in the system response, we begin with an explanation of the anti-resonance phenomenon observed by Lysyansky et al. [20]. First, consider the case of chronic CRS with a sequential stimulation site activation order (Fig 2D). Note that in the cluster state regime, the value of *σ* is low, so the spatial stimulation decay profile is narrow and there is little overlap in stimulation from adjacent stimulation sites (Fig 3A). That is, whenever a particular oscillator is stimulated, it is stimulated by the same site every time. Further, recall that the CR period *T* is chosen to be equal to the mean natural period of the oscillator network, such that a given stimulation site is always active at the same relative mean phase (modulo 2π). For example, stimulation site 1 is always active when the mean phase (Fig 2E) is $\psi_{1}(t)=\Omega t\in [0,\frac{2\pi }{N_{c}}),mod\;2\pi$. This type of tightly localized in-phase periodic stimulation entrains the sub-group of oscillators to the periodic bursting of its corresponding stimulation site. As a result, we have *N*_*c*_ sub-groups of oscillators, evenly spaced around the unit circle, with subgroup *j* oscillating at the mean natural frequency *Ω* and a phase offset of $j\frac{2\pi }{N_{c}},j=1,\ldots ,N_{c}$. Since the clusters are evenly spaced, we see a resultant low value of *R*_*1*_ and a high value of *R*_*4*_ (since *N*_*c*_ = 4).

Now additionally consider the ON-OFF CRS pattern used by Lysyansky et al. [20], where the entire stimulation is ON for *m* periods of length *T*, and OFF for *n* periods (Fig 2C). As before, the stimulation sites are activated in sequential order, each for a duration of $\frac{T}{N_{c}}$. At the beginning of each ON period, the order is reset so the sequence re-starts at stimulation site 1. Notice that the regions of optimal *first-order* desynchronization performance, i.e. low *<r>*_*1*_, occur along the diagonals where (*m+n*) takes on an integer value (Fig 5C). These regions correspond to high values of *<r>*_*4*_ (Fig 5D), indicative of the clustering behavior described above. The performance is worst and clustering does not occur whenever $(m+n)=k+\frac{1}{2}$, where *k* is an integer.

Taking a closer look at the stimulation paradigm for a non-integer case (*m*+*n* = 3.75) (Fig 2C), we see that a given stimulation site no longer activates at the same mean phase (mod 2π) of the oscillator network (Fig 2E), every time. Rather, the activation time relative to the mean phase changes every ON cycle. For example, stimulation site 1 is active when $\psi_{1}(t)\in [0,\frac{2\pi }{N_{c}}),mod\;2\pi$ during the odd ON cycles, but when $\psi_{1}(t)\in [\pi ,\;\frac{2\pi }{N_{c}}+\pi ),mod\;2\pi$ during the even ON cycles. As a result, the sub-group of oscillators cannot become entrained to the periodic bursting of its respective stimulation site, since it does not consistently burst at the same relative phase. Therefore, clustering does not occur and we do not see a suppression of *R*_*1*_.

Whenever (*m+n*) is an integer, however, the ON-OFF period *(m+n)T* is an integer multiple of the mean natural oscillator period $\frac{2\pi }{\Omega }=T$, so the start of every ON period occurs whenever the mean phase *ψ*_1_(t) = Ω*t* = 0,mod 2*π*. Therefore, if a stimulation site is active, it is active at the same relative mean phase every time (modulo 2π). As in chronic CRS, four evenly spaced clusters are created, resulting in a high value of *<r>*_*4*_ and low *<r>*_*1*_.

In contrast, during periodic flashing, the ON-OFF pattern is independent of the contact cycling order. As a result, whenever a stimulation site is active, it is guaranteed to be active at the same relative mean phase (modulo 2π) as it would be during chronic CRS. Thus, we see strong suppression of the first order parameter (Fig 5A) and consistent clustering behavior (Fig 5B) regardless of the value of *m* and *n*. It is, of course, necessary that enough total stimulation is delivered, as seen in the lower right corner of the figures where the ON periods are very short and OFF periods are long, and suppression of synchrony does not occur. Note that whenever (*m+n*) is an integer, the periodic flashing paradigm is identical to the ON-OFF paradigm used by Lysyansky et al. [20].

The previous discussion suggests that activation of stimulation sites at the same relative phase in every cycle is essential for clustering which, in turn, causes first-order desynchronization. To verify this idea we consider a third ON-OFF paradigm, where the activation order of stimulation sites is randomly permuted every CR period, while the flashing overlay *F(t)* remains the same as before (Fig 2B). As expected, we fail to see any clustering behavior and poor suppression of first-order synchrony. *<r>*_*1*_ is high (Fig 5E) and *<r>*_*4*_ is low (Fig 5F) for all values of *m* and *n*. Note that in this limit, the paradigm used by Lysyansky et al. and periodic flashing are effectively equivalent.

### Uniform desynchronization

**Fig 6:**
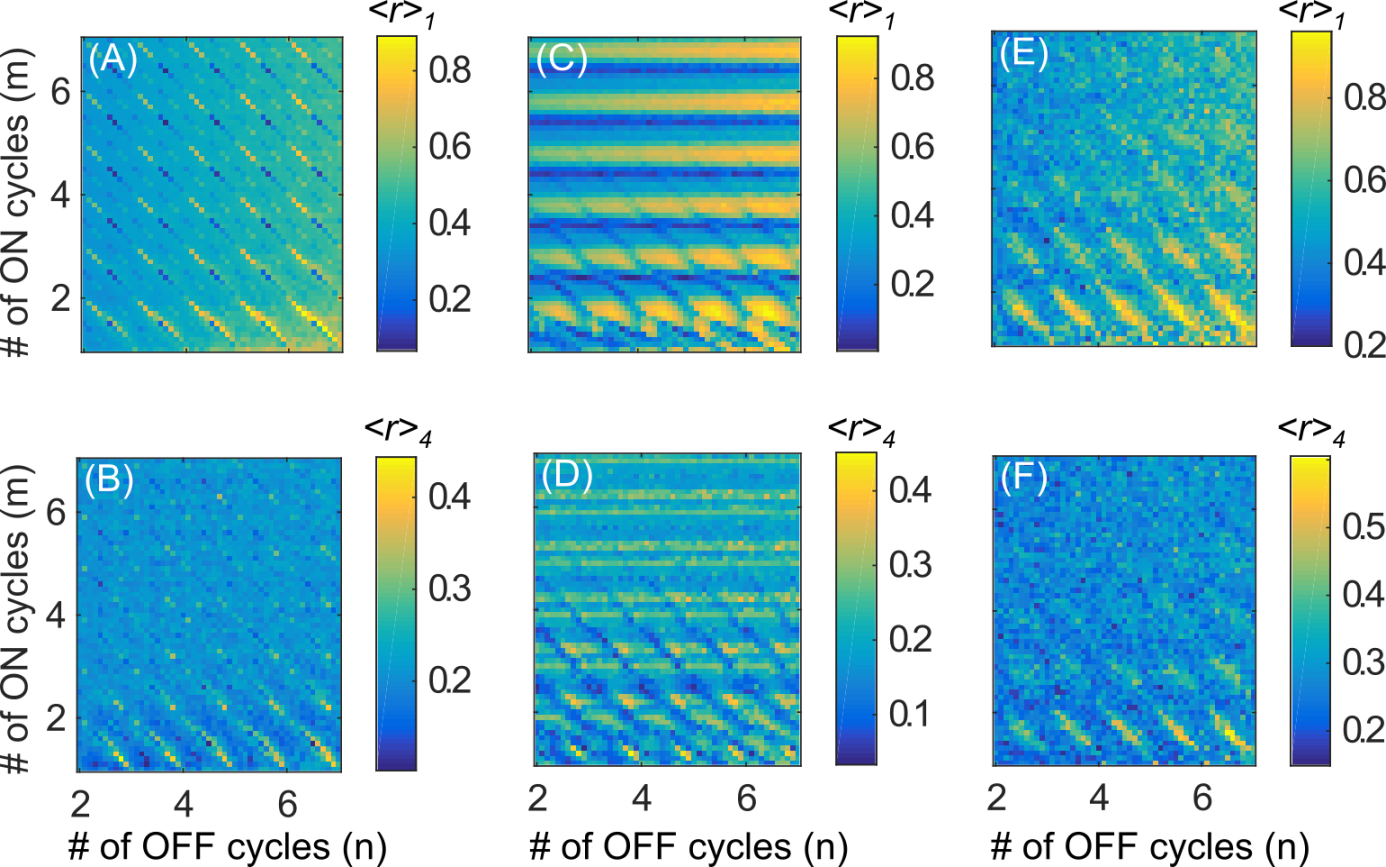
Mean maximum of the order parameter as a function of *m* and *n* for different stimulation paradigms in the desynchronization state regime (*I* = 7, *σ =* 2). Subplots as in Fig 5.

We next explore the performance of the periodic flashing paradigm in the “desynchronization state” regime (*I* = 7, *σ =* 2). Although we do not see optimal desynchronization performance, periodic flashing does achieve acceptable intermediate results, and successfully removes the dependence on the ON and OFF periods (Fig 6A), with the exception of the apparent thin diagonal stripes which are addressed below. In contrast, when using the paradigm proposed by Lysyansky et al. [20], we see a different structure in the plot of *<r>*_*1*_ as a function of *m* and *n*: alternating horizontal regions of good and poor performance (Fig 6C). Note that this structure, although less prominent, is also present during the clustered state regime (Fig 5C).

First, note that the anti-resonance phenomenon is entirely absent in this regime, since the clustering mechanism that causes anti-resonance does not exist. The spatial stimulation spread profile is much wider (Fig 3B), so all the oscillators are significantly stimulated by all four stimulation sites. As a result, lacking tight spatial localization of stimulation, a cluster state cannot be achieved in this regime and we see low values of *<r>*_*4*_, regardless of *m* and *n*, or the stimulation paradigm (Fig 6B, 6D and 6F).

To understand the cause of the horizontal stripes, following the analysis of Lysyansky et al. [20], we consider the behavior of the oscillator network immediately after the CR stimulation is turned off, as a function of *t*_*off*,_, the time at which it is turned off (modulo the CR period *T*). We stimulate the network with chronic CRS until *R*_*1*_*(t)* reaches a stable periodic state (no more than 5 CR periods when using the parameters in Table 1) and then turn off the stimulation part-way through the next CR period. Recall that since *K* > *K*_*c*_, the unstimulated network will spontaneously approach a synchronized state, i.e. *R*_*1*_*(t)* → 1.

**Fig 7:**
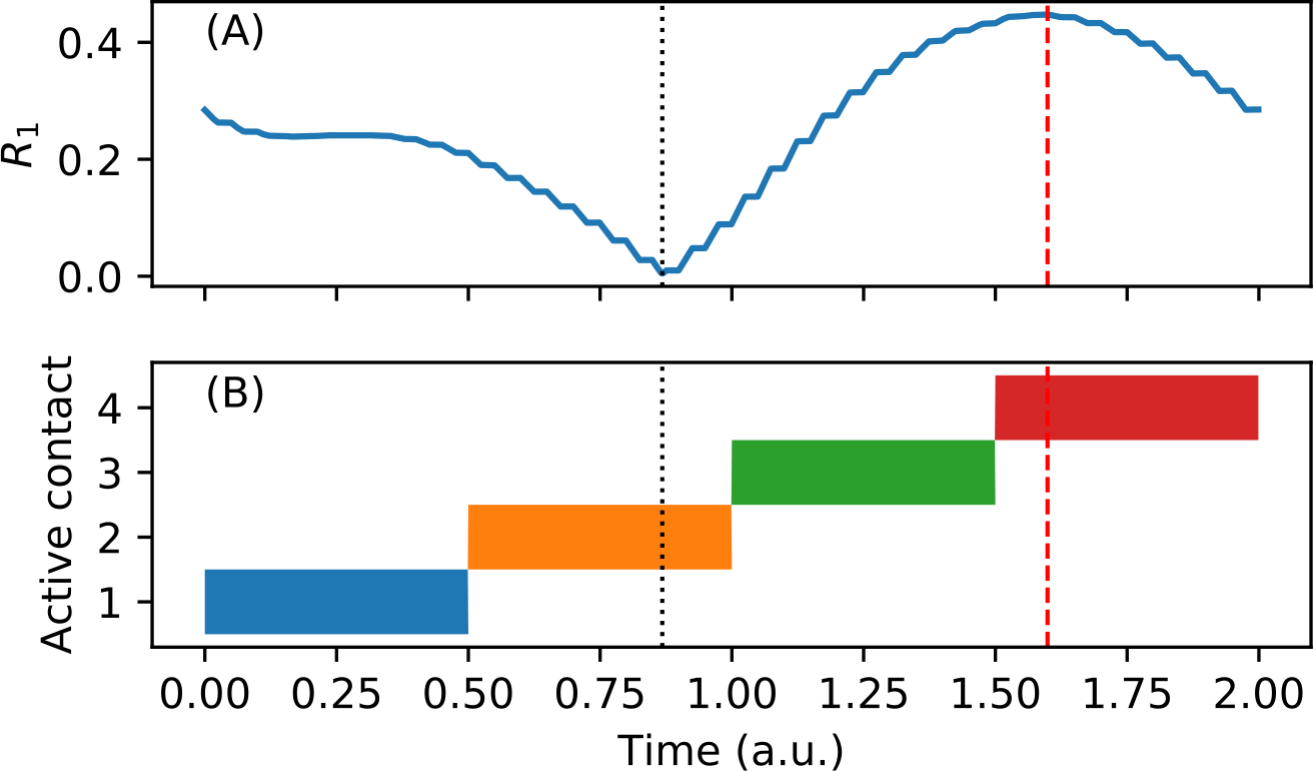
Optimal time to turn off stimulation depends on the magnitude of the first order parameter at that time. (A) First order parameter during one CR period (*T* = 2) in the desynchronization state regime (*I* = 7, *σ =* 2), after reaching steady-state. Optimal *t*_*off*_ occurs when *R*_*1*_ is minimal, indicated by dotted black vertical line. Converseley, pessimal *t*_*off*_ occurs when *R*_*1*_ is maximal, indicated by dashed red line. (B) Periodic CRS pattern that induces the time-varying order parameter shown in A. Optimal *t*_*off*_ occurs at a fixed point within the stimulation site activation pattern. In this example, it is always approximately 0.38 time units after the activation of the second stimulation site.

Naturally, if *R*_*1*_ is smaller when the stimulation is turned off, then the transient will be longer. Since we would like to maintain a desynchronized state for as long as possible, the optimal *t*_*off*_ is whenever *R*_*1*_ is at the minimum, i.e. $t_{\mathrm{opt}}=\underset{0\leq t<T}{\mathrm{argmin}}\;R_{1}(t)$. Conversely, the pessimal *t*_*off*_ is when *R*_*1*_ is maximal. In the desynchronization state regime, *t*_*opt*_ ≈ 0.88 (Fig 7A) and in the cluster state regime *t*_*opt*_ ≈ 0.53. Since chronic CRS is periodic with period *T*, after reaching steady-state *R*_*1*_*(t)* is also periodic with the same period. Consequently, *t*_*opt*_ also corresponds to a particular time point in stimulation site activation pattern. For example, in the desynchronization state regime, *t*_*opt*_ occurs 0.38 time units after the second stimulation site is activated (Fig 7B).

We now consider the effect of *t*_*off*_ during ON-OFF CRS. Recall that in the paradigm used by Lysyansky et al. [20], stimulation site 1 is always the first one to be activated in any given ON period, and thus all the ON periods end at the same time point within the stimulation site activation pattern. Therefore, the length of the ON cycle, *m*∙*T*, is effectively equal to *t*_*off*_. As expected, we see low *<r>*_*1*_ when *m*∙*T* ≈ *t*_*opt*_ ≈ 0.88 (mod *T*) and high *<r>*_*1*_ when *m*∙*T* ≈ 1.6 (mod *T*) (Fig 6C, cf. Fig 7). For very small *m*, since the network does not fully reach steady state within the ON period, there is an added dependency on *n*. Note that this structure is less prominent in Fig 5A because the difference between *R*_*1*_ at the optimal and pessimal *t*_*off*_ is smaller in the cluster state regime than that in the desynchronization state regime (Fig 7A).

In contrast, with periodic flashing, we see that the dependence on *m* is almost completely eliminated, with the exception of the diagonals (*m+n*) = *k*, where *k* is an integer (Fig 6A). Also note that the value of *<r>*_*1*_ seen in most of Fig 6A is approximately mid-way between the maximum and minimum values in the figure. This averaging effect occurs because the ON-OFF flashing function *F(t)* is independent of the stimulation site cycling functions *ρ*_*j*_*(t)* and therefore *t*_*off*_ is at a different place within the activation pattern for each new ON period. Over many ON periods, *t*_*off*_ ranges between the optimal and the pessimal time and so $r_{1}^{\left(i\right)}$ in (6) also ranges between the lowest and highest it could be. As a result, we see an intermediate value of *<r>*_*1*_ for most values of (*n*,*m*).

As noted in the previous section, along the diagonals defined by (*m+n*) = *k*, the periodic flashing paradigm is identical to the ON-OFF paradigm used by Lysyansky et al. [20]. Within these diagonals, during any given ON period, *t*_*off*_ is at the same place within the stimulation site activation pattern. So, we see low *<r>*_*1*_ only when *t*_*off*_ *= m*∙*T* ≈ 0.88 (mod *T*), since the behavior along the diagonals is governed by a single *t*_*off*_. Similarly, in the cluster state regime, the optimal-*t*_*off*_ structure is suppressed, except along the diagonals for which (*m+n*) is an integer (Fig 5A and 5C).

For completeness, as in the previous section, we further consider an ON-OFF paradigm with a randomized order of stimulation site activation (Fig 2B). We see a washing out of the prominent diagonals seen under sequential-cycling periodic flashing (Fig 6A and 6E), particularly for larger values of *m* (e.g. *m*>3). This is to be expected since randomizing the stimulation site activation order breaks the periodicity of the CRS pattern, and therefore also the periodicity of *R*_*1*_*(t)*. As a result, *t*_*opt*_ is different in every ON period and so we get a similar averaging effect we saw in the (*m+n*) ≠ *k* regions of Fig 6A. Note that we do not see a total suppression of the diagonals since there is still a temporal organization to the CRS pattern–the stimulation site switches every $\frac{T}{N_{c}}$ time units which induces a weak temporal structure in *R*_*1*_*(t)*. Therefore *t*_*opt*_ is not uniformly distributed within the ON period and the diagonals, although much less prominent, are still present.

## Discussion and conclusions

The initial motivation behind CRS was to drive the leading order parameter *R*_*1*_ to zero by inducing a symmetric clustered state [13,14]. The remaining order parameters are then also driven to zero due to the slaving principle [36]. The resulting uniformly desynchronized state exhibits the greatest possible degree of desynchronization and as a result it has been considered as the goal of neuromodulation. Because the resultant uniformly desynchronized state is unstable, the network eventually resynchronizes if left undisturbed. To maintain the desynchronized state, CRS was either delivered with a demand-controlled timing (i.e. whenever *R*_*1*_ exceeded a pre-defined threshold) or demand-controlled pulse trains (i.e. the length of the tonic stimulation bursts was adapted to *R*_*1*_) [13–17]. A single CR stimulus turned out to induce the longest and most pronounced transient uniform desynchronization if the phases of the stimulated subpopulations were symmetrically spaced on the unit circle, e.g. in case of four stimulation sites, if a symmetric 4-cluser state with large *R*_*4*_ and *R*_*1*_ close to zero was achieved [13,14].

Due to technical constraints and, in particular, with a focus on theoretically predicted long-lasting effects [15], the closed-loop, demand-controlled CRS delivery protocols were approximated by periodic protocols, with periodic sequences of either 4:2 [23] or 3:2 [24–26] ON-OFF cycles. The intention behind these open-loop protocols is to let the unperturbed network run though a transient uniformly desynchronized state during the OFF cycles. For a pre-defined threshold of *R*_*1*_ optimal relationships between integer *m* and *n* were determined for the ON-OFF CRS paradigm used by Lysyansky et al. [20]. If one performs chronic CRS (without OFF cycles) and terminates CRS at different phases of the last CR cycle, the duration and strength of the transient desynchronization may depend on that phase [20]. This motivated Lysyansky et al. to take into account an ON-OFF CRS protocol with non-integer *m* and *n* combined with an ON period-triggered restart of the CR stimulus pattern (Fig 2C), demonstrating the sensitivity of the protocol to the duration of the ON and OFF periods.

In this paper, we have introduced a novel CRS paradigm called *periodic flashing* and explored its effect on desynchronization in the context of two stimulation regimes that achieve qualitatively different synchronization states–high intensity, spatially focused stimulation creating a “cluster state” and low intensity, spatially broad stimulation creating a “desynchronization state.” The previously studied CRS paradigm [20] exhibits sensitivity to the precise choice of ON and OFF periods in either regime through anti-resonance in the “cluster state” regime, or the need for precisely tuned shut-off times in the “desynchronization state” regime. In contrast, the periodic flashing paradigm does not exhibit this sensitivity and leads to desynchronization of the synchronized oscillator network for a wide range of ON and OFF periods in both regimes.

Our results also suggest that, although on average the desynchronization state regime is slightly worse at suppressing *R*_*1*_ than the cluster state regime, the desynchronization state regime is likely more biologically relevant than the cluster state regime for this specific computational model. Previous clinical and preclinical studies have all used ON and OFF periods that are integer multiples of the underlying pathological frequency. However, these studies also used a *randomized* stimulation site ordering (Fig 2B) [24–26] and found, apart from pronounced long-lasting effects achieved only with CR-DBS, an acute reduction of symptoms comparable to those seen with traditional DBS. Assuming that these DBS equivalent outcomes are an indication of significant desynchronization, these experimental observations appear at odds with the effect observed in the cluster state regime, where a sequential ordering (Fig 2A) is far more effective than randomized ordering at suppressing synchrony (Fig 5A and 5E). On the other hand, in the desynchronization state regime we do not see a significant drop in performance when switching from sequential-cycling (Fig 6A) to randomized-cycling (Fig 6E) periodic flashing, suggesting that the desynchronization state regime is a more biologically plausible one, given the model we are using.

The Kuramoto model is very abstract and there are, of course, limitations. These can be addressed by increasing the complexity at the expense of computational time. For instance, this model is noise-free and we may choose to add a noise term *ξ*_*i*_*(t)* to the right hand side of (1) [13–15]. Spike-timing dependent plasticity is a factor that may strongly affect the long-term behavior of the model, which can be implemented by replacing the coupling constant *K* with a time-dependent value for each pair of oscillators as in [15]. Furthermore, in practice, the distribution of natural frequencies, as measured by the local field potential (LFP), is much more broad and multi-modal [26]. This can be addressed by using a more complicated distribution of the frequencies in (1). Finally, we may use a more biologically detailed model such as [37] or [38], although such models may be computationally tractable only for exploring small subsets of the parameter space. We leave these studies for future work.

As an avenue for further exploration, periodic flashing may be generalized to other “flashing” variations. One example is “randomized flashing” where the ON and OFF periods do not have a fixed duration. That is, the number of ON cycles or OFF cycles, or both, may be randomly chosen at the start of each ON-OFF period. Alternatively, with “time-varying flashing” the ON and OFF periods may vary deterministically over time. A further generalization is to perform the “flashing” of each contact independently, either in a fixed, time-varying, or random fashion. Even more broadly, any of these flashing variations can be used in conjunction with other stimulation site activation patterns. The pattern can be any fixed or time-varying pattern, or a randomized one as we have explored in this work. The randomization itself can be varied by using different probability distributions of stimulation site activation. Furthermore, in all of these paradigms, the stimulation site activation is not restricted to binary activation of one site at a time. Two or more sites may be active simultaneously, with varying amounts of power delivered by each one.

Finally, while this paper only discusses open-loop stimulation protocols, increasingly over the past few years, interest has grown in closed-loop DBS. In the context of “flashing” CRS, closed-loop setups might enable novel approaches for the control of abnormal neuronal synchrony. Dynamic characteristics of neuronal activity may vary, e.g., within different phases of motor tasks [39,40] or between different movement and/or freezing states [41]. According to our computational findings, within appropriate CRS parameter ranges, the flashing approach provides flexibility concerning the ON-OFF timing of CRS: the time windows with CRS ON need no longer be linked to the fundamental CRS periodicity. Future studies may be devoted to demand-controlled or, in general, any type of closed-loop flashing. For instance, flashing might enable to perform sensing during appropriate time windows in order to regularly or occasionally recalibrate fundamental CRS parameters. By a similar token, flashing might adjust CRS to relevant physiological processes, e.g., by avoiding stimulation during potentially vulnerable time windows. A variety of candidate biomarkers and control schemes for controlling closed-loop stimulation have been proposed [42–46]. However, the viability of these proposed biomarkers remains a topic of debate [47,48]. Exploring these issues remains a topic for future research.

## Supporting information

S1 Code Python 2.7 libraries for simulating coordinated reset stimulation in a Kuramoto oscillator network. (ZIP)
